# Therapeutic role of memantine for the prevention of cognitive decline in cancer patients with brain metastasis receiving whole-brain radiotherapy: a narrative review

**DOI:** 10.1590/1980-5764-DN-2021-0102

**Published:** 2022-05-23

**Authors:** Melmar Cerilo Folloso, Mazelle Torres, Stephen Lowell Ciocon, Jed Noel Ong, Jerickson Abbie Flores, Joseree-Ann Catindig

**Affiliations:** 1 Jose R. Reyes Memorial Medical Center, Department of Neurology, Manila, Philippines.; 2 Jose R. Reyes Memorial Medical Center, Department of Radiotherapy, Manila, Philippines.

**Keywords:** Memantine, Radiotherapy, Brain neoplasms, Cognition, Memantina, Radioterapia, Neoplasias encefálicas, Cognição

## Abstract

Brain metastases are the most common central nervous system tumors. The mainstay treatment for this tumor in low to middle income countries is whole brain radiation therapy. Irreversible cognitive decline is associated with the use of whole brain radiotherapy. Several pharmacologic and nonpharmacologic options have been employed in studies focusing on the prevention of cognitive decline following whole-brain radiation therapy. Memantine use has been shown to provide some benefit in reducing the rate of decline in cognitive function and time to cognitive failure. The objective of this review article is to provide a summary on available primary literature on the therapeutic role of memantine for the prevention of cognitive decline in cancer patients with brain metastasis receiving whole brain radiotherapy.

## RESUMO.

As metástases cerebrais são os tumores mais comuns do sistema nervoso central. O tratamento principal para este tumor em países de baixa e média renda é a radioterapia de cérebro inteiro. O declínio cognitivo irreversível está associado ao uso de radioterapia cerebral total. Várias opções farmacológicas e não farmacológicas têm sido empregadas em estudos com foco na prevenção do declínio cognitivo após radioterapia de cérebro inteiro. O uso de memantina demonstrou fornecer algum benefício na redução da taxa de declínio na função cognitiva e no tempo até a falha cognitiva. O objetivo deste artigo de revisão foi fornecer um resumo da literatura primária disponível sobre o papel terapêutico da memantina para a prevenção do declínio cognitivo em pacientes com câncer com metástase cerebral recebendo radioterapia cerebral total.

## INTRODUCTION

Metastases to the brain are the most common central nervous system tumors. It occurs in 20-40% of all patients with malignant tumors (mostly from lung and breast cancers)^1^. There has been an increasing trend toward survival among these patients and this may be due to improved diagnostic modalities and improvement in treatment regimens. The incidence of brain metastasis is estimated to be about 17,000 per year in the United States, which is 10 times higher than the incidence of the most common primary brain tumors^2^.

A study in 2016 revealed that the incidence of brain neoplasms in the Philippines is 2297, with 1969 deaths^3^. A retrospective study in 2015 done in a tertiary hospital showed metastatic brain disease accounted for 3.2% of all central nervous system neoplasms^4^. In our institution, a retrospective chart review identified 86 patients with metastatic brain disease. Currently, there are no existing national registries for brain tumors and attempts have been made at an institutional level.

## METHODS

The records were searched until December 30, 2020, and identified through PubMed, Embase, ClinicalTrials.gov, ICTRP (WHO), and Cochrane Library databases. The following search strategy was implemented, and these key words (in the title/abstract) were used: “Memantine” AND “Cognitive Dysfunction” AND “Brain metastasis” AND “Radiotherapy” OR “Whole Brain Radiotherapy.” The search strategy was used to obtain the titles, abstracts, and, if necessary, the full text of articles of the relevant studies in English, and they were independently screened to determine the suitability. The reference lists of the studies were also reviewed to ensure literature saturation.

The inclusion criteria were as follows: (1) primary research studies including adult patients with brain metastases; (2) studies evaluating radiation therapy, including whole-brain radiation therapy (WBRT) or stereotactic radiosurgery (SRS) either alone or in combination, as initial or postoperative treatment, with or without systemic therapy (immunotherapy and chemotherapy); (3) studies comparing eligible interventions to other eligible interventions or other management approaches; (4) studies reporting on the following outcomes: overall survival, progression-free survival recurrence/cancer control, symptom burden, and health status or health-related quality of life; (5) studies including national and international settings; and (6) all randomized controlled trials (RCTs), prospective experimental and observational studies. The exclusion criteria were as follows: (1) study samples comprising patients with primary brain tumors and done on pediatric samples; (2) studies without WBRT treatment arms; (3) unavailability of results, different study population, and different intervention; (4) partial result information and duplicate studies; (5) reviews, commentaries, viewpoints, or opinions; and (6) animal studies.

Initial search strategies were done by MOT and MCF. Disagreement was decided by a third reviewer, who was either JNO, JAC, or JAF.

**Figure 1.**
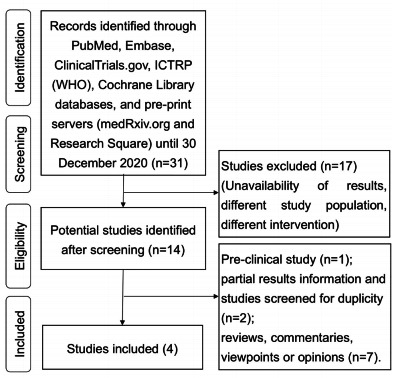
Flowchart depicting the steps of qualitative synthesis of evidence from the literature.

The following data were extracted from the included studies: author (year), study design, level of evidence, sample size, inclusion criteria, study arms, outcome, and result of outcome. The search strategy is presented in Figure 1. Table 1 presents the summary of the studies included in the article.

**Table 1.** Summary of the clinical studies on the effect of memantine for the prevention of cognitive dysfunction in patients with brain metastasis receiving whole-brain radiation therapy.

Author (year)	Study design	Level of evidence	n^1^	Inclusion criteria	Study arms	Outcome	Result of outcome
Brown et al. (2013)^26^	Randomized double-blind, placebo-controlled trial	1	554	Adult patients with brain metastases receiving WBRT^2^	WBRT+memantine WBRT+placebo	HVLT-R for Delayed Recall at 24 weeks	No significant difference in delayed recall (primary outcome) between the two arms p=0.059)
Brown et al. (2020)^28^	Randomized parallel, open-label controlled trial	1	518	Adult patients with brain metastases outside a 5-mm margin around either hippocampus receiving WBRT	WBRT+memantine HA-WBRT^4^+memantine	Time to cognitive function failure	Significant reduction in cognitive failure in patients under HA-WBRT plus memantine (adjusted hazard ratio, 0.74; 95%CI 0.58-0.95; p=0.02).
Wong et al. (2016)^27^	Randomized parallel, open-label placebo-controlled trial	1	14	Adult patients with brain metastases receiving WBRT (12 from RTOG 0614)	WBRT+placebo WBRT+Memantine*	DCE-MRI measures of tumor tissue and normal-appearing white matter (NAWM) vascular permeability	significantly (p=0.01) reduced normal-appearing vascular permeability changes following radiotherapy
Laack et al. (2018)^25^	Randomized parallel, open-label controlled trial	1	442	Adult patients with brain metastases receiving WBRT (from RTOG 0614)	WBRT+memantine WBRT+placebo	Association of health-related quality of life and cognitive function	Baseline cognitive function correlated significantly with Medical Outcomes Scale-Cognitive Functioning Scale (MOS-C).

*One patient did not receive any drug; WBRT: whole-brain radiation therapy.

## TREATMENT FOR BRAIN METASTASES

The treatment for brain metastasis is individualized depending on the primary cancer, the patient’s clinical history, and the number of metastases. The blood-brain barrier protects the brain and is only permeable to limited substances, rendering tumors located in this area difficult to treat with conventional medical therapies^5^ ^,^ ^6^. Surgery has been performed in single lesions^7^; however, radiation therapy remains the most used treatment modality, especially in low- to middle-income countries (LMICs).

Whole-brain radiation therapy remains the primary therapeutic tool for patients with brain metastases^8^ ^,^ ^9^ ^,^ ^10^. About 100,000 patients with brain tumor who received brain irradiation survive for >6 months and 50-90% of these patients exhibit disabling cognitive dysfunction^11^. Attention has been directed toward neurocognitive decline which affects learning, memory, processing speed, attention, and executive function. The mechanisms of radiation-induced cognitive decline are similar to those seen in vascular dementia patients, and this includes radiation-accelerated atherosclerosis, mineralizing microangiopathy, followed by vascular insufficiency and infarction^12^.

The exact mechanism by which WBRT induces cognitive dysfunction is still not fully understood, but brain injury may be due to injuries in different cell types. Currently, there are several hypotheses by which WBRT may cause cognitive dysfunction. One hypothesis is the significant reduction of neurogenesis in the hippocampus^12^. An experimental study done on rats showed >95% reduction in new neuron production following a single dose of WBRT. The authors further added that there two important ways by which neurogenesis is reduced. First, radiation-induced damage to nasopharyngeal cancers (NPCs) impairs growth potential of the progenitor pool following long-term treatment as exemplified above. Second, radiation may induce changes in the brain microenvironment, leading to prominent inflammatory response. This would lead to activation of microglia, thus impairing neurogenesis^13^.

Radiation may also alter the brain’s microvasculature that maintains hippocampal neurogenesis. The exact mechanism is still being debated, but studies have shown that disruption caused by the radiation may lead to decreased size in the perivascular clusters of precursor cells. This change may last for several years and may be responsible for the reduction in neurogenesis after completion of cranial radiation therapy^12^ ^,^ ^13^.

**Figure 2.**
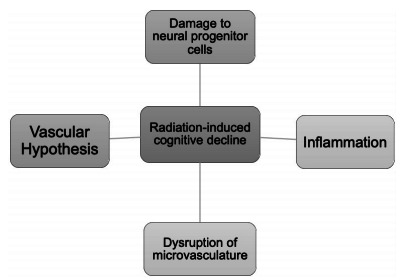
Potential mechanisms in radiation-induced cognitive decline and the role of memantine.

Another hypothesis regarding radiation-induced cognitive dysfunction is the vascular hypothesis. It states that the vascular changes seen in post-radiation patients are similar to those seen in cases of vascular dementia. Mechanism includes death of endothelial cells and increased platelet adhesion, leading to thrombus formation. This would eventually result in occlusion of small vessels. In addition, there may also be increased atherosclerosis, ultimately leading to vascular ischemia and/or infarction. Ischemia or infarction may increase the levels of glutamate, the principal excitatory neurotransmitter in the brain^12^. In normal physiological conditions, glutamate activates the NMDA receptors to enhance learning and store memory. However, in diseased conditions, the excessive increase in glutamate could lead to increased neurotoxicity^14^. This mechanism may serve as a target for therapy in radiation-induced cognitive toxicity. These mechanisms are summarized in Figure 2.

Despite the importance and clear concern about radiation-induced cognitive decline, the pathophysiology driving the progression of this syndrome remains poorly understood.

The pathophysiology of radiation-induced cognitive decline is still not fully understood. For this reason, there is great interest in studying treatments to prevent or reduce radiation-induced cognitive injury.

## CURRENT OPTIONS FOR THE PREVENTION OF COGNITIVE DECLINE

### Stereotactic radiosurgery

Several treatment options are in current practice for preventing radiation-induced cognitive decline. One such option is SRS. SRS is a procedure that safely delivers high doses of radiation to a defined target and this modality has been studied in a number of clinical trial. An RCT showed the efficacy of SRS alone in lowering the risk of significant decline in learning and memory function when compared to the combined SRS+WBRT^15^. Among patients with one to three lesions, SRS alone again showed significantly less cognitive decline at 3 months when compared to combined SRS+WBRT (-28.2% difference, p<0.001) despite no difference in overall survival^16^. Among postoperative patients, the NCCTG N107C/CEC·3^17^ trial revealed that there are more frequent cognitive decline in patients receiving WBRT compared to SRS, with no difference in overall survival. A total of 194 patients were randomly assigned to both arms with a median follow-up time of 11.1 months. Primary outcome is cognitive-deterioration-free survival. This was longer in the SRS group compared to patients with WBRT (HR=0.47, p<0.001.

### Hippocampal avoidance

The hippocampus has been identified as a key player in the process of learning and memory^18^. Several strategies were formulated to primarily avoid this part of the brain during radiation therapy. The RTOG 0933^19^ is a single-arm phase II multicenter trial that investigated the concept of avoiding the hippocampus during WBRT. It revealed that there is significantly lower decline in Hopkins Verbal Learning Test-Revised for Delayed Recall (HVLT-R DR) in comparison with a historical control group at 4 months from baseline (mean decline of 7.0 vs. 30% in the control group, p<0.001).

### Pharmacological options

Pharmacological options were also explored. Donepezil is an acetylcholinesterase inhibitor currently used in the treatment of Alzheimer’s disease. A randomized placebo-controlled trial^20^ revealed that a single daily dose of donepezil (5 mg for 6 weeks and 10 mg for 18 weeks) did not significantly improve verbal learning, memory, and other composite scores. The authors further added that increasing the dose in future trials may be of greater benefit for patients. Another option is armodafinil, a drug primarily used in the treatment of narcolepsy. A study^21^ showed that the drug was well tolerated but had no significant effect on fatigue and cognitive function when given during radiation therapy. Methylphenidate is another option. It is a stimulant used for the treatment of attention-deficit hyperactivity disorder (ADHD). A randomized trial showed that the drug did not significantly improve the quality of life and cognitive outcome measures in patients on radiation therapy.

### Memantine

Memantine hydrochloride (MEM) is an indicated treatment for moderate-to-severe dementia of the Alzheimer’s type. It has neuroprotective properties and is used as off-label to treat Parkinson’s disease, chronic brain syndrome, and spasticity^22^. MEM is a low-affinity uncompetitive antagonist of NMDA and thus displaced rapidly, thereby avoiding its negative consequences on memory. MEM only interacts with the receptor in pathological conditions, such as in radiation-induced cognitive dysfunction and Alzheimer’s disease^23^. ­Microglial NMDA receptors are present and may cause inflammatory responses during overactivation. This inflammatory response is mediated by factors such as interleukins, TNF, ROS, and nitric oxide. This mechanism may be another way by which MEM may help in protecting against cognitive dysfunction^23^ ^,^ ^24^. ­Memantine was safe and well tolerated and reduced the risk of cognitive decline, as measured by several standard screening tests^25^.

## EFFECT OF MEMANTINE ON RADIATION-INDUCED COGNITIVE FUNCTION

A landmark trial by Brown et al.^26^ in 2013 evaluated the protective effects of memantine in radiation-induced cognitive dysfunction. This is a randomized, double-blind, placebo-controlled trial that included 508 individuals with confirmed brain metastases by contrast-enhanced magnetic resonance imaging (MRI). Other inclusion criteria include Karnofsky performance status of ≥70, stable disease within 3 months prior to the study, normal serum levels of creatinine, total bilirubin, and blood urea nitrogen (BUN). Participants are also required to have a Mini-Mental State Examination (MMSE)>18 with no allergy on memantine. Patients with prior treatment such as radiosurgery and surgical resection was included, given the therapy >14 days prior to the start of the study. The primary outcome is cognitive function after 24 weeks as measured by the HVLT-R DR.

The participants were allocated via the Zelen treatment allocation scheme and received either placebo or memantine for 24 weeks within 3 days of the start of radiation therapy. Each subject received escalating doses of memantine starting at 5 mg daily dose to a target daily dose 20 mg at week 4 and maintained until 24 weeks. For the WBRT, each subject received a total dose of 37.5 Gy composed of 15 fractions of 2.5 Gy. ­Assessment was done at baseline, 8, 16, 24, and 52 weeks after the commencement of the study. This included clinical history, neurological and physical examination, specimen collection, and neuropsychological battery of tests.

Participants were majority female, with 55.1 and 57.5% in the treatment and control groups, respectively. The median age is 60 years for the memantine group and 59 years in the placebo group. Notably, 70% of the subjects have lung cancer as its primary disease site. In terms of neurological functional status, 44.9 and 38.9% are having minor symptoms but fully active in the memantine and control groups, respectively. The primary cognitive outcome for this study was not significant despite having less decline in HVLT-R DR in the memantine arm compared with the placebo arm at 24 weeks (median decline of 0 vs. -0.9). The authors noted that the high attrition rate and low number of subjects analyzed contributed to the non-significant result, having a low 35% statistical power. Other cognitive tests showed statistical significance, including the raw score of the MMSE (median decline 0 vs. -1, p=0.009). Time to cognitive failure was found to have significantly favored the memantine group, with a 21% relative risk reduction. The effect of steroids during treatment was also evaluated and showed that patients treated with steroids had more decline at 8 weeks of treatment. Overall survival and progression had no statistically significant difference between the two arms.

The authors concluded that memantine is well tolerated and safe among these patients. They added that it showed significance in terms of reducing the rate of cognitive decline and the time to cognitive failure despite not having a significant result in their primary outcome. This study is limited by the poor compliance of its participants due to factors such as tumor progression and death. The study also did not include participants with a low Karnofsky score; hence, the benefit of memantine on these patients is still unknown.

Another study done by Wong et al.^27^ in 2016 evaluated the ability of dynamic contrast-enhanced MRI (DCE-MRI) in detecting vascular changes in patients receiving WBRT and memantine. There were 14 patients included in this trial, 12 of whom are from RTOG 0614. The primary outcome measure is the normal-appearing white matter area under the curve (NAWM AUC) measured at different time points (8, 16, and 24 weeks) after WBRT. Cognitive and quality-of-life assessment was also done. Each arm had the same number of subjects. In this study, the most common primary site is the lung, followed by the breast.

The patients on memantine therapy had significantly lower AUC at 6 months post-WBRT compared to placebo (p=0.01). The treatment group had better cognitive functions than those on placebo (p=0.03). However, there was no significant difference in the overall survival rates between groups. The study concluded that the results suggest the value of memantine in reducing vascular changes seen in WBRT patients.

## RECENT AND FUTURE TRIALS

Recent studies used memantine as their standard of care. One study by Brown et al.^28^ in 2020 evaluated the impact of hippocampal avoidance WBRT in preserving cognitive function. The risk for cognitive failure was significantly lower in HA-WBRT plus memantine versus WBRT plus memantine alone as (adjusted HR=0.74; p=0.02). Despite not evaluating the effectiveness of memantine alone in preventing cognitive dysfunction, the trial established this combination as the new standard of care in the setting of WBRT. This would become a reference for future studies.

Unfortunately, studies on memantine are almost exclusively on patients with brain metastases receiving WBRT and it has not been evaluated in pediatric patients and other brain tumors. The SPiRiT (ClinicalTrials.gov Identifier: NCT04567251)^29^ trial is a randomized, placebo-controlled, double-blind study evaluating the role of memantine in improving cognitive function in adult cancer survivors who received prior brain irradiation regardless of tumor type. Two studies (i.e., ClinicalTrials.gov Identifier: NCT03194906 and ClinicalTrials.gov Identifier: NCT04217694)^30^ ^,^ ^31^ are currently evaluating the impact of memantine on pediatric patients.

## SUMMARY

Although memantine is not used as a standard of care in the clinical setting across all patients receiving WBRT treatment for brain metastases, recent literature supports that memantine use is safe, well-tolerated, and may have benefit in reducing the rate of cognitive decline. This may be more beneficial among patients who survived longer. The effect may be due decreasing vascular changes post-WBRT treatment. Furthermore, ongoing clinical trials are now using memantine as a standard of care and evaluating its effect in other tumors and in the pediatric population. Future studies could focus on the economic viability of memantine, especially on LMICs.
